# Adenoviral Mediated Expression of BMP2 by Bone Marrow Stromal Cells Cultured in 3D Copolymer Scaffolds Enhances Bone Formation

**DOI:** 10.1371/journal.pone.0147507

**Published:** 2016-01-25

**Authors:** Sunita Sharma, Dipak Sapkota, Ying Xue, Yang Sun, Anna Finne-Wistrand, Ove Bruland, Kamal Mustafa

**Affiliations:** 1 Department of Clinical Dentistry, Faculty of Medicine and Dentistry, University of Bergen, Bergen, Norway; 2 The Gade Laboratory for Pathology, Department of Clinical Medicine, University of Bergen, Bergen, Norway; 3 Department of Fibre and Polymer Technology, Royal Institute of Technology, Stockholm, Sweden; 4 Department of Medical Genetics and Molecular Medicine, Haukeland University Hospital, Bergen, Norway; Georgia Regents University, UNITED STATES

## Abstract

Selection of appropriate osteoinductive growth factors, suitable delivery method and proper supportive scaffold are critical for a successful outcome in bone tissue engineering using bone marrow stromal cells (BMSC). This study examined the molecular and functional effect of a combination of adenoviral mediated expression of bone morphogenetic protein-2 (BMP2) in BMSC and recently developed and characterized, biodegradable Poly(L-lactide-co-є-caprolactone){poly(LLA-co-CL)}scaffolds in osteogenic molecular changes and ectopic bone formation by using *in vitro* and *in vivo* approaches. Pathway-focused custom PCR array, validation using TaqMan based quantitative RT-PCR (qRT-PCR) and ALP staining showed significant up-regulation of several osteogenic and angiogenic molecules, including *ALPL* and *RUNX2* in ad-BMP2 BMSC group grown in poly(LLA-co-CL) scaffolds both at 3 and 14 days. Micro CT and histological analyses of the subcutaneously implanted scaffolds in NOD/SCID mice revealed significantly increased radiopaque areas, percentage bone volume and formation of vital bone in ad-BMP2 scaffolds as compared to the control groups both at 2 and 8 weeks. The increased bone formation in the ad-BMP2 group *in vivo* was paralleled at the molecular level with concomitant over-expression of a number of osteogenic and angiogenic genes including *ALPL*, *RUNX2*, *SPP1*, *ANGPT1*. The increased bone formation in ad-BMP2 explants was not found to be associated with enhanced endochondral activity as evidenced by qRT-PCR (*SOX9* and *FGF2*) and Safranin O staining. Taken together, combination of adenoviral mediated BMP-2 expression in BMSC grown in the newly developed poly(LLA-co-CL) scaffolds induced expression of osteogenic markers and enhanced bone formation *in vivo*.

## Introduction

Bone regeneration using tissue engineering approach, which utilizes mesenchymal stem cells grown in an osteoconductive scaffold and with osteoinductive growth factors is a promising approach and has been studied for over a decade [1]. Bone Morphogenetic protein-2 (BMP2), a member of a TGF-β superfamily is a potent osteoinductive growth factor. BMP2 promotes cellular migration, proliferation and osteogenic differentiation of mesenchymal stem cells during bone repair by regulating the expression of Runt-related transcription factor 2 (Runx2) and Osterix (Osx) [2]. The FDA approved recombinant BMP2 (rhBMP2) protein has been utilized in numerous preclinical and clinical studies for osteogenesis [3, 4]. Delivery of BMP2 to the defect site is crucial for its biological activity. Owing to its short half-life and rapid diffusibility from the applied site, supraphysiological dose is required to achieve biologic effects, making it an expensive treatment strategy [5]. In addition, use of supraphysiological dose is associated with side effects such as: heterotropic ossification [6], osteolysis [7] and structurally abnormal bone formation [8]. This highlights the need for a more physiological and efficient alternative approach for growth factor delivery. Recently, gene delivery technique, where a therapeutic gene is delivered into a defect site by transgenes employing viral or non-viral vectors, has gained a considerable interest in bone tissue engineering (BTE) [9–11].

Another important component in BTE is the supportive scaffold in which the bone marrow stromal cells (BMSC) attach, proliferate, differentiate and release signaling molecules and growth factors. An ideal scaffold is required to be biocompatible, biodegradable, must be able to promote cellular interactions and bone formation, and have adequate mechanical and physical properties [12, 13]. In this regard, scaffolds of various materials [including metals, ceramics, polymers (natural or synthetic) or their combination] have been tested for their applicability in BTE. Ceramic-based scaffolds, such as tricalcium phosphate and hydroxyapatite, resemble the inorganic bone matrix and have a good osteoconductive property. However, wider clinical application of these materials have been hindered by their slow degradation rate and brittleness [14], and limited ability to stimulate cell differentiation [15]. The recently developed poly(LLA-co-CL) scaffolds have been suggested to possess excellent biodegradability and biocompatibility qualities required for BTE [16, 17]. These scaffolds have been extensively characterized for the biological and molecular response of osteoblasts [18], endothelial cells [19] as well as human-BMSC and rat-BMSC [20, 21]. These copolymer scaffolds can be tailored to achieve predictable degradation rate as per the various clinical need and application [12]. Owing to this ability, copolymer scaffold can also be an ideal candidate to be used as a delivery device for various growth factors including rhBMP2. Mechanical and surface characteristics of these scaffolds have been successfully modified to enhance bone regeneration [22]. Both non-covalent (physiosorption, physical entrapment) and covalent binding techniques have been used for the delivery of rhBMP2 utilizing these copolymer scaffolds to accelerate bone regeneration in critical sized bone defects [23, 24]. Nevertheless, osteogenic ability of BMSC engineered to express BMP2 by adenoviral system grown in poly(LLA-co-CL) scaffolds has not been tested and characterized for use in BTE. In the current study, using BMSC grown in poly(LLA-co-CL) scaffolds, we showed that adenoviral mediated expression of BMP2 in BMSC is associated with significantly enhanced osteogenic ability both *in vitro* and *in vivo*.

## Materials and Methods

### Cell Culture

Well characterized primary human BMSC were purchased from StemCell Technologies (Cat. number: MSC-001F, Vancouver, British Columbia, Canada) and expanded using MesenCult Proliferation Kit (StemCell Technologies, Part ID 05411) following standard culture protocol. All cell culture experiments were carried out at a humidified atmosphere of 37°C and 5% CO_2_. For validation of the osteogenic and angiogenic gene expression changes induced by adenoviral mediated BMP2 expression in BMSC, commercially available human BMSC from two additional donors were purchased and used (henceforth referred to as donor 2 and 3 BMSC) as described in S1 Text.

### Preparation of BMSC seeded scaffolds

Poly(LLA-co-CL) scaffolds were fabricated, using the solvent-casting particulate-leaching method as described previously [16, 21]. For *in vitro* experiments, scaffolds (diameter ≈12mm, height≈1.3mm, porosity: 85% and pore size: 350μm on average) were placed on the bottom of 48-well plates, pre-wetted with the culture media and incubated overnight at a humidified atmosphere of 37°C and 5% CO_2_. BMSC were seeded at a density of 5 × 10^4^ cells/scaffold.

### Adenoviral expression vector construction and transduction of BMSC

Replication-deficient adenoviral expression vector carrying the coding sequences of *BMP2* gene (reference sequence: NM_001200.2)(ad-BMP2)and *eGFP* gene coding for enhanced green fluorescent protein (eGFP) was purchased from Cyagen Biosciences Inc. Adenoviral vector carrying only *eGFP*coding sequences (ad-GFP) was used as a control. Adenoviral particles were generated by transfecting HEK 293 cells (ATCC-CRL-1573) with Pac I digested constructs. Early passage (passage 2–3) BMSC were infected as monolayer culture with respective adenoviruses (multiplicity of infection, MOI = 100 to 150) to obtain 80–90% infection efficiency as examined under the fluorescent microscope. BMSC infected with ad-BMP2 and ad-GFP will henceforth be referred to as ‘ad-BMP2 BMSC’ and ‘ad-GFP BMSC’ respectively. To verify up-regulation of *BMP2* mRNA in ad-BMP2 BMSC in monolayer, cells were harvested after 48 hours of adenoviral infection. To determine the amount of secreted BMP2 after 48 hours of adenoviral infection, culture supernatant was collected and analyzed using commercially available ELISA kit following manufacturer’s instructions. Further, BMSC were seeded at a density of 5 × 10^4^ cells/scaffold after 48 hours of infection in monolayer with the respective adenoviral particles in Poly(LLA-co-CL) scaffolds. BMSC grown in scaffolds were harvested after 3 and 14 days for mRNA expression analysis. Culture supernatants were also collected on the respective time points for ELISA assay.

### Total RNA extraction

Total RNA from the *in vitro* seeded scaffolds were extracted using Maxwell^®^ 16 LEV simplyRNA Kit (Cat no: AS1270, Promega) on a Maxwell^®^ 16 instrument following the manufacturer’s protocol. Quantity and purity of the total RNA was determined using a Nanodrop Spectrophotometer (ThermoScientific Nano Drop Technologies, Wilmington, DE, USA). Agilent 2100 Bio analyzer (Agilent Technologies) was used to examine the integrity of RNA (data not shown).

### Expression analysis of osteogenesis and angiogenesis related genes *in vitro*

To examine the range of genes modulated by BMP2 over-expression in BMSC in the 3D-scaffold (*in vitro*), a custom PCR array (Cat no: 330131, SuperArray Bioscience, Frederick, MD, USA) containing primer pairs for 30 genes related to osteogenesis and angiogenesis (Table 1) was used. Total RNA from 3 biological replicates (*n* = 3) of both ad-GFP and ad-BMP2 groups were used for cDNA synthesis at 3 and 14 days. PCR amplification was performed using the following cycling conditions: 95°C for 10 min, (95°C for 15 sec, and 60°C for 1 min) x 40 cycles in ABI Prism Sequence Detector 7900 HT (Applied Biosystems, Foster City, USA). Pre- and post- PCR quality control measures, as recommended by the manufacturer, were strictly followed. PCR array data were analysed as described previously [25]. Briefly, threshold cycle (Ct) was used to calculate 2^-ΔCt^ value for each gene using PCR Array Data Analysis Web Portal (SABiosciences). 2^-ΔCt^ values were then exported to microarray data analysis software (J-Express 2012). For statistical analysis, unsupervised hierarchical clustering and significance analysis of microarray (SAM) tests were used. Differentially expressed genes with false discovery rate (FDR) = 0 were considered to be significantly modulated genes.

**Table 1:** Genes used in the custom qRT-PCR array.

Gene Symbol	Refseq #	RT2 Catalog Number
*ANG*	NM_001145	PPH00376
*ANGPT1*	NM_001146	PPH00374
*ANGPT2*	NM_001147	PPH00377
*FGF1*	NM_000800	PPH00067
*FGF2*	NM_002006	PPH00257
*FLT1*	NM_002019	PPH00375
*KDR*	NM_002253	PPH00386
*PGF*	NM_002632	PPH01155
*VEGFA*	NM_003376	PPH00251
*COL4A3*	NM_000091	PPH02131
*PECAM1*	NM_000442	PPH01362
*HIF1α*	NM_001530	PPH01361
*MMP9*	NM_004994	PPH00152
*MMP2*	NM_004530	PPH00151
*BGLAP*	NM_199173	PPH01898
*BMP2*	NM_001200	PPH00549
*BMP4*	NM_130851	PPH00546
*BMP6*	NM_001718	PPH00542
*BMP7*	NM_001719	PPH00527
*COL1A1*	NM_000088	PPH01299
*RUNX2*	NM_004348	PPH01897
*SPP1*	NM_000582	PPH00582
*ALPL*	NM_000478	PPH01311
*ITGB1*	NM_002211	PPH00650
*FN1*	NM_002026	PPH00143
*SOX9*	NM_000346	PPH02125
*FST*	NM_006350	PPH01954
*BMPR1A*	NM_004329	PPH01929
*BMPR2*	NM_001204	PPH00401
*GAPDH*	NM_002046	PPH00150
*ACTB*	NM_001101	PPH00073
*B2M*	NM_004048	PPH01094

### Reverse transcription and quantitative RT-PCR (qRT-PCR) using TaqMan assays

Three hundred Nano grams of total RNA was converted to cDNA by reverse transcription reaction using a high capacity cDNA Archive Kit (Applied Biosystems, Carlsbad, CA, USA). *BMP2* TaqMan assay (Hs00154192_m) was used to verify the expression of *BMP2* mRNA in adenovirus transduced BMSC. The *in vitro* PCR array results were independently validated by performing qRT-PCR using TaqMan assays for selected key genes {*ALPL* (Hs01029144_m1), *RUNX2*(Hs00231692_m1), *BGLAP* (Hs01587814_g1) and *VEGFA* (Hs00900055_m1)}. *GAPDH* (Hs99999905_m1) was used as an endogenous control. All qRT-PCR amplifications were performed on ABI Prism Sequence Detector 7900 HT (Applied Biosystems, Foster City, USA) with standard cycling conditions. Comparative 2^-ΔΔ Ct^ method was used to quantify the relative mRNA expression.

### Alkaline phosphatase (ALP) staining

ALP staining was done to analyze the osteoblastic differentiation potential of BMSC transduced with ad-BMP2. After 48 hours of infection, cells were trypsinized and 2 × 10^4^ cells were seeded in monolayer on 4-well culture dish. ALP staining was done on day 3, and 14. Briefly, cells were washed with phosphatase buffer saline and stained for alkaline phosphatase (ALP) activity using Napthol AS-TR phosphate and fast red violet B salt (Sigma-Aldrich) as described previously [26].

### *In vivo* subcutaneous implant model in nonobese severe combined immunodeficient (NOD/SCID) mice

#### a. Preparation of BMSC seeded scaffolds

Poly(LLA-co-CL) scaffolds (diameter ≈ 6mm, height≈1.3mm and pore size of 90–500μm) were prepared as described previously [16, 21]. The scaffolds were placed on the bottom of 96-well plates, pre-wetted with the culture medium and incubated overnight at a humidified atmosphere of 37°C and 5% CO_2_. 5 × 10^5^ BMSC were seeded in each scaffold after 48 hours of infection with the respective adenoviral particles, incubated overnight at a humidified atmosphere of 37°C and 5% CO_2_ and implanted subcutaneously in NOD/SCID mice. Scaffolds with untransduced BMSC or without any BMSC (only scaffold) were used as additional controls in the *in vivo* experiments.

#### b. Surgical Implantation of scaffolds

All animal experiments were approved by the Norwegian Animal Research Authority and conducted according to the European Convention for the Protection of Vertebrates used for Scientific Purposes, with local approval number 4940. Sixteen NOD-SCID mice (6–8 weeks old, Taconic Farms, Denmark) (8 mice each for both 2 and 8 week time-points) were used for subcutaneous implantation of scaffolds. The animals were anesthetized by subcutaneous injection Midazolam 5mg/ml/Hyponorm solution. Two midline surgical incisions of approximately 2cm in length were made on the back of mice, which were extended laterally by blunt dissection and a subcutaneous pouch was created. Each animal received four randomly allocated scaffold implants of the following groups: i) ad-GFP (9 replicates), ii) ad-BMP2 (9 replicates), iii) untransduced (9 replicates) and iv) scaffold only group (5 replicates). The scaffolds were implanted in such a way that scaffolds from all the experimental groups are implanted in all possible locations among following four sites: upper right side, upper left side, lower left side and lower right side. Wounds were closed with Histoacryl Tissue Adhesive (*n*-butyl cyanoacrylate) (3M, St. Paul, MN, USA). Animals were euthanized with CO_2_ inhalation and subsequent cervical dislocation at 2 and 8 weeks. Three of the randomly selected scaffolds from only scaffold group and six scaffolds each from un-transduced, ad-GFP and ad-BMP2 groups at 2 week time point were divided into two equal halves. One half of the scaffolds was stored in RNA later (Ambion) for total RNA extraction and the other half was formalin fixed (4% buffered formalin), decalcified (12.5% EDTA and 2.5% PFA in phosphate buffered saline) and paraffin embedded for histological analysis. Remaining scaffolds from all of the experimental groups were also formalin fixed and used for histological analysis. At 8 weeks, 3 randomly selected scaffolds from all of the groups were used for micro CT analysis. All remaining scaffolds were formalin fixed-paraffin embedded for histological analysis.

### Expression analysis of osteogenesis and angiogenesis related genes for *in vivo* scaffold explants

For *in vivo* scaffold explants (2 weeks), six hundred Nano grams of total RNA from 6 biological replicates (*n* = 6) of both ad-GFP and ad-BMP2 BMSC groups were converted to first strand cDNA using RT^2^ First Strand Kit (C-03, SABiosciences, Frederick, MD, USA). To examine the range of genes modulated by BMP2 over-expression, a custom PCR array (Cat no: 330131, SuperArray Bioscience, Frederick, MD, USA) containing primer pairs for 30 genes related to osteogenesis and angiogenesis (Table 1) was used. PCR amplification and statistical analysis was performed as described beforehand for the *in vitro* scaffolds.

### Histological analysis

The paraffin embedded specimens were cut into 5 μm sections. The sections were deparaffinized, rehydrated in xylene and graded ethanol stained with Hematoxylin and Eosin. Safranin O staining was performed to visualize newly synthesized cartilaginous extracellular matrix glycosaminoglycan. Briefly, sections were stained with 0.1% Safranin O and 0.05% fast green was used as counter stain. The stained sections were dehydrated, cleared and mounted using resinous medium.

### Micro computed tomography analysis (μCT)

The *in vivo* scaffold explants harvested at 8 weeks were scanned with a SkyScan 1172 X-ray μCT imaging system (Aartselaar, Belgium) at 10 μm resolution with a voltage of 60 kV with 0.5 aluminum filters. The Projection image was reconstructed using Nrecon software. The quantitative analysis of the image was performed by CTan software provided by SkyScan. A global threshold of 70–255 was applied to all the samples and the region of interest (ROI) was selected by outlining the scaffolds. The percent of binarized volume within ROI represented the percent of bone volume in the scaffolds.

### Statistical analyses

Data are expressed as mean ± standard error of the mean (SEM). Statistical analysis for the comparison of means between two groups was performed with Student’s *t*-test; whereas ANOVA test with Bonferroni post hoc analysis was used for comparison between multiple groups. Statistical analyses were performed using GraphPad Prism software version 5.00 for Windows (GraphPad Software, San Diego California USA, www.graphpad.com), with the level of significance set at 5%.

## Results

### BMP2 adenoviral expression vector up-regulated endogenous *BMP2* mRNA and secreted protein levels

**Fig 1:**
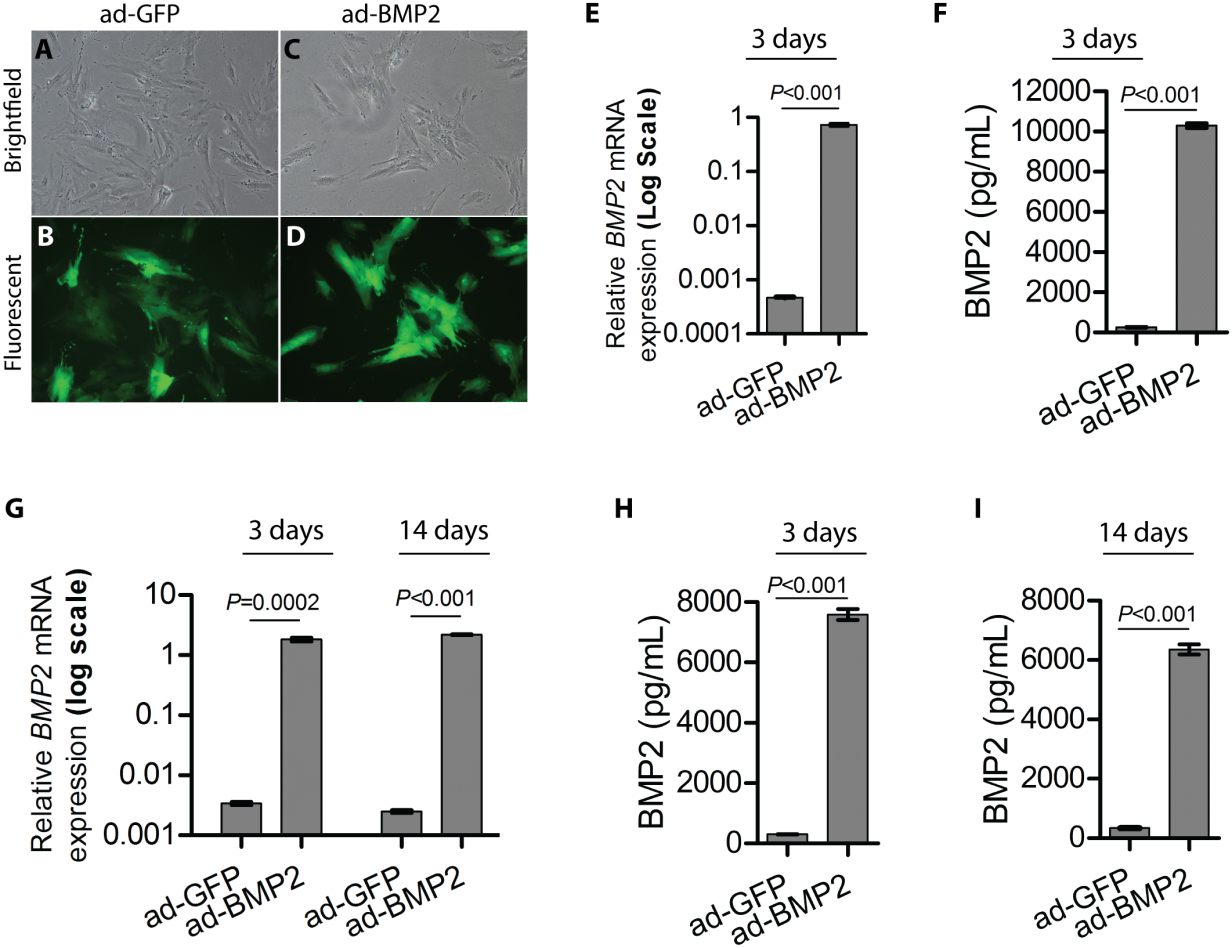
BMP2 adenoviral expression vector increased up-regulation of BMP2 in BMSC. Representative images of BMSC in monolayer transduced with ad-GFP (A, brightfield and B, fluorescent) and ad-BMP2 (C, brightfield and D, fluorescent) adenoviral particles. Significant up-regulation of *BMP2* mRNA (E) and secreted BMP2 protein in the conditioned medium (F) at day 3 in ad-BMP2 BMSC in monolayer as compared to the ad-GFP BMSC. Error bars in (E) represent SEM of 3 repeated experiments (*n* = 3) done in 3 technical replicates. Student’s-t test was performed for statistical analysis. Error bars in (F) represent SEM of 3 repeated experiments. Student’s *t*-test was performed for statistical analysis. (G) 5× 10^4^ BMSC were seeded in each poly(LLA-co-CL) scaffolds and harvested after 3- and 14-days for qRT-PCR. qRT-PCR demonstrated significant up-regulation of endogenous *BMP2* mRNA in ad-BMP2 BMSC as compared to the ad-GFP BMSC both at 3- and 14- days. *BMP2* mRNA level was normalized to *GAPDH* mRNA level. Error bars represent SEM of 3 repeated experiments (*n* = 3) done in 3 technical replicates. Student’s *t*-test was performed for statistical analysis. (H-I) Higher levels of secreted BMP2 were observed by using ELISA assay in the culture supernatant of ad-BMP2 BMSC as compared to the ad-GFP transduced cells both at 3- and 14- days. Error bars in (H and I) represent SEM of 3 repeated experiments. Student’s *t*-test was performed for statistical analysis.

BMSC transduced with ad-GFP and ad-BMP2 viral particles demonstrated similar morphology in monoculture (Fig 1A–1D). ad-BMP2 BMSC grown in monolayer expressed significantly higher levels of *BMP2* mRNA and secreted BMP2 protein at day 3 as compared to the control ad-GFP BMSC (Fig 1E and 1F). In parallel, significantly higher levels of *BMP2* mRNA and secreted BMP2 protein was found in ad-BMP2 BMSC grown in 3D scaffold as compared to the control ad-GFP transduced cells both at day 3 and 14 (Fig 1G–1I).

### BMP2 over-expression is associated with up-regulation of osteogenic and angiogenic markers *in vitro*

**Fig 2:**
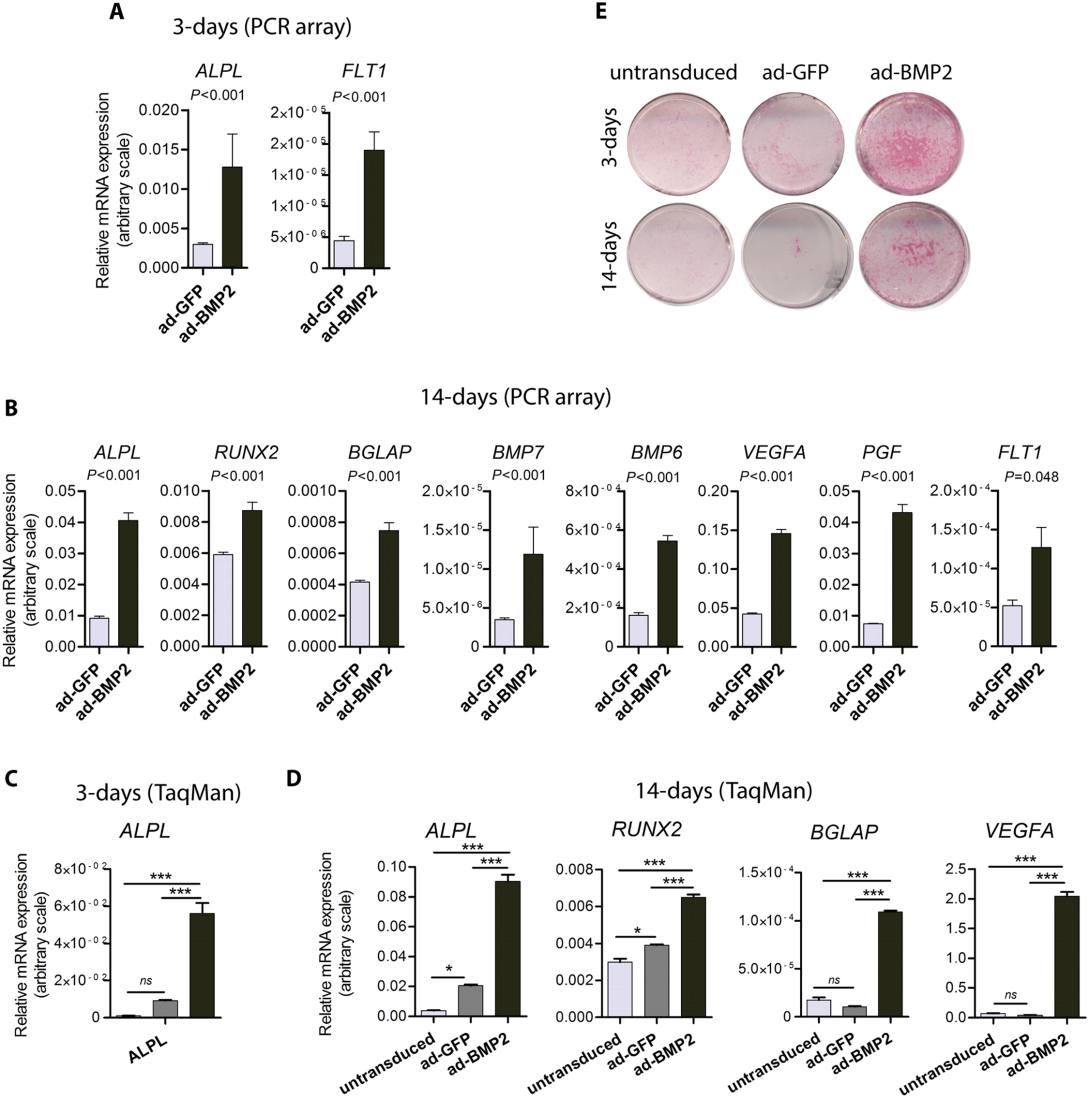
BMP2 over-expression led to up-regulation of osteogenic and angiogenic molecules in vitro. 5 × 10^4^ ad-BMP2 or ad-GFP BMSC were seeded in each Poly(LLA-co-CL) scaffolds and harvested after 3 and 14 days for custom PCR array and TaqMan based qRT-PCR. (A) With SAM analysis, *ALPL* and *FLT1* mRNA levels were found to be significantly (FDR = 0) up-regulated at 3-days in ad-BMP2 BMSC. (B) More osteogenic and angiogenic markers were significantly induced at 14-days in ad-BMP2 BMSC as compared to the ad-GFP BMSC group. (C and D) Array results for *ALPL*, *RUNX2*, *BGLAP* and *VEGFA* genes were independently validated in ad-BMP2 BMSC by using TaqMan qRT- PCR. Error bars represent SEM of 3 repeated experiments (*n* = 3) done in 3 technical replicates. ANOVA test with Bonferroni post hoc analysis was performed for statistical analysis in C and D. ***, *p*<0.001; *, *P*<0.01; ns, non-significant. (E) Representative images demonstrating higher alkaline phosphatase activity in ad-BMP2 BMSC in monolayer as compared to the ad-GFP BMSC and untransduced BMSC. Experiments were repeated at least three times.

Custom PCR array was used to examine differentially expressed osteogenesis and angiogenesis related genes with BMP2 over-expression. SAM analysis showed significant (FDR = 0) up-regulation of the early osteogenic marker *ALPL* at day 3 in ad-BMP2 BMSC grown in 3D-scaffols as compared to the control ad-GFP BMSC (Fig 2A). At day 14, *ALPL*, *RUNX2*, *BMP6*, *BGLAP* (osteocalcin) and *BMP7*, the osteogenesis related genes, were found to be over-expressed in ad-BMP2 BMSC (Fig 2B). Additionally, mRNA expression levels of angiogenic molecules, such as *FLT1* (VEGFR1) and its ligands *VEGFA* and *PGF*, were significantly upregulated in ad-BMP2 BMSC respectively at 3 days and 14 days (Fig 2A and 2B). Independent validation of the differentially expressed selected genes, as identified by PCR array, was done by performing qRT-PCR using TaqMan assays for *ALPL*, *RUNX2* and *BGLAP* and *VEGFA*. Consistent with the PCR array results, *ALPL* was found to be significantly up-regulated in ad-BMP2 BMSC and untransduced BMSC both at day 3 (*P*<0.001) and day 14 (*P*<0.001) (Fig 2C and 2D). Similarly, mRNA levels of *RUNX2* (*P*<0.001), *BGLAP* (*P*<0.001) and VEGFA (*P*<0.001) were significantly higher in ad-BMP2 as compared to that of ad-GFP BMSC and untransduced BMSC at day 14 (Fig 2D). To confirm the induction of *ALPL* mRNA at the protein level, ALP staining was performed. ALP level was found to be significantly induced at day 3 and the elevated level was also maintained at day 14 in ad-BMP2 BMSC as compared to the barely detectable levels in ad-GFP BMSC and untransduced BMSC (Fig 2E). Further, over-expression of mRNA levels of *ALPL*, *RUNX2* and *VEGFA* with adenoviral mediated BMP2 over-expression was confirmed in two additional donors as described in S1 Text and S1 Fig.

### BMP2 over-expression induced bone formation in 3D-scaffold explants in NOD/SCID mice

**Fig 3:**
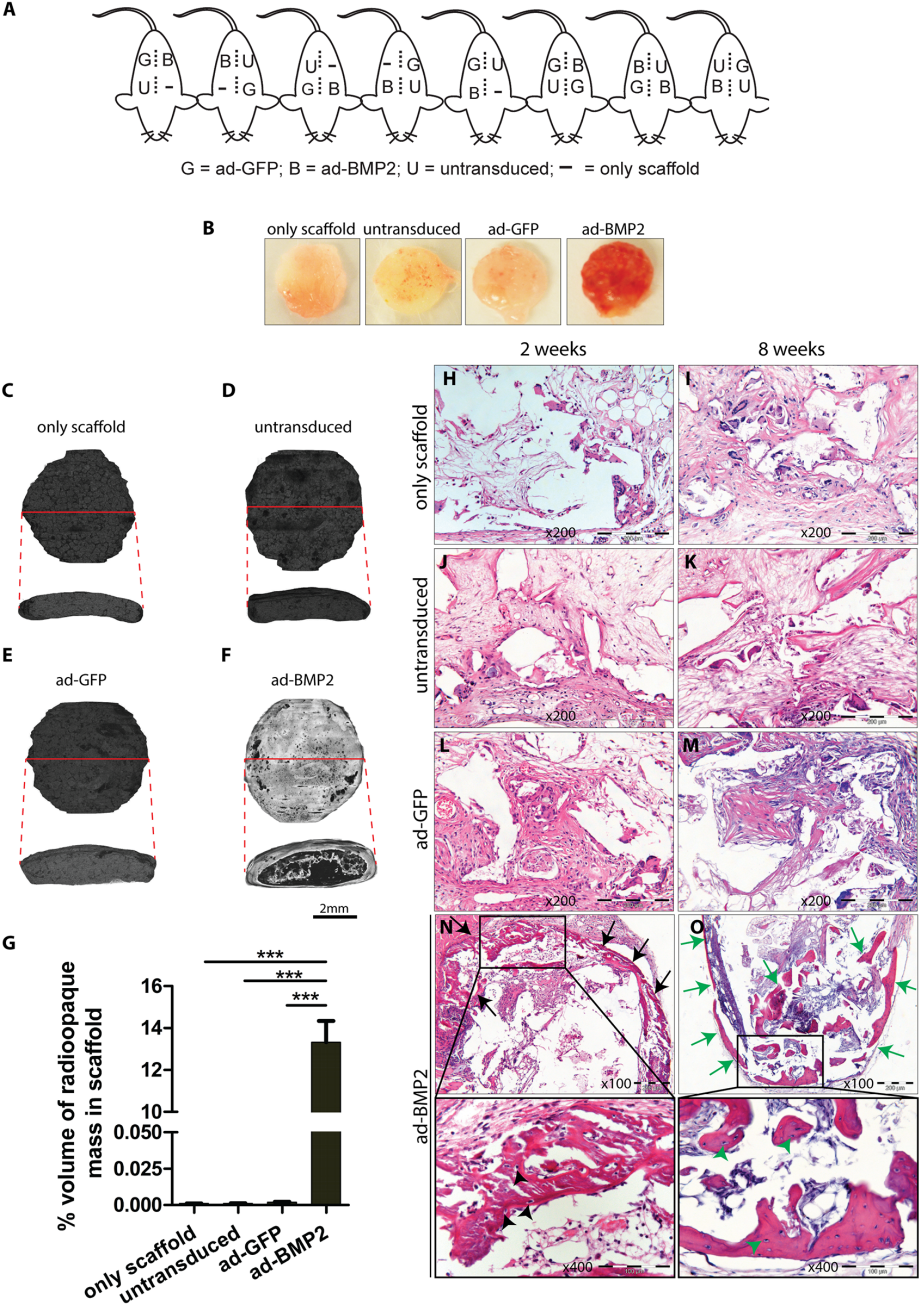
Over-expression of BMP2 induced ectopic bone formation in poly(LLA-co-CL) scaffold explants in subcutaneous NOD/SCID mice model. Ability of BMP2 to induce ectopic bone formation in poly(LLA-co-CL) scaffold was examined by subcutaneously implanting scaffold seeded with 5 ×10 5 ad-BMP2 BMSC or control BMSC in NOD/SCID mice. (A) Schematic illustration indicating location of the scaffold implantation sites in the back of each mouse. (B) Representative images of the scaffold explants at 8 weeks revealed more vascularity in ad-BMP2 BMSC as compared to the control explants. (C-F) To evaluate the ectopic bone formation, scaffold explants at 8 weeks were examined by micro CT. No radiopaque bone like object was detected in the control groups (C), (D) and (E). However, radiopaque bone like areas were found at the periphery as well as inside of the scaffold in ad-BMP2 explants (F). Red line across the scaffold indicates imaginary plane for transverse section. (G) Quantification of radiopaque bone like objects in the scaffold explants at 8-weeks demonstrated significant amount of bone formation in ad-BMP2 scaffolds. ANOVA test with Bonferroni post hoc analysis was performed for statistical analysis in (G). Error bars represent SEM. ***, *P*<0.001. (H-O) Representative images of H & E stained formalin fixed paraffin embedded sections of scaffold explants from different experimental groups at 2- and 8-weeks. (H-M) Formation of bony structures was not detected in only scaffold, untransduced or ad-GFP groups both at 2- and 8-weeks. (N) As early as 2-weeks, formation of bony structures (black arrows) mostly at the periphery of the scaffold explants was observed in ad-BMP2 BMSC group. (O) Extensive bone formation (green arrows) with bony trabeculae extending throughout the thickness of the Scaffolds was found in 8-weeks in ad-BMP2 BMSC. Bony structure consisted of numerous osteocyte like cells both at 2- (black arrowheads, inset N) and 8- weeks (green arrowheads, inset O).

Ability of BMP2 to induce ectopic bone formation in 3D-scaffold was examined by subcutaneously implanting 3D-scaffold seeded with ad-BMP2, ad-GFP, untransduced BMSC and only scaffold controls (Fig 3A). With tactile and visual inspection, ad-BMP2 scaffold explants were found to be harder in consistency with richer vascular networks as compared to that of the control-explants both at 2 and 8 weeks (data for 2 weeks are not shown) (Fig 3B). Micro CT analysis revealed no radiopaque bone like structures in the scaffold explants from only scaffold (*n* = 3), untransduced (*n* = 3) and ad-GFP (*n* = 3) groups at 8 weeks (Fig 3C–3E). In contrast, all analyzed replicates (*n* = 3) from ad-BMP2 explants at 8 weeks revealed formation of dense bone like structure in the periphery as well as inside of the scaffold explant (Fig 3F). Quantification of ectopic bone formation in the scaffolds showed significantly higher % volume of radiopaque mass in ad-BMP2 explants as compared to the controls (Fig 3G). Histological examination of H & E stained sections was next performed to confirm the formation of bone structures in ad-BMP2 explants. Similar to the microCT findings, no bony structures were detected in all replicates of only scaffold, untransduced and ad-GFP explants both at 2 and 8 weeks (Fig 3H–3M). However, formation of bony structures (black arrows) was detected at the periphery of the scaffold explants in all replicates (9/9) of ad-BMP2 BMSC at 2 weeks (Fig 3N). At 8 weeks, formation of bony structures (green arrows) was more extensive with bony trabeculae extending throughout the whole thickness of the scaffold explants in all of replicates (6/6) examined (Fig 3O). Bony structure consisted of numerous osteocyte like cells both at 2 (black arrowheads, inset N) and 8 (green arrowheads, inset O) weeks. No inflammatory cells, except occasional multinucleated giant cells, were seen in all groups (Fig 3H–3O).

### BMP2 over-expression mediated bone formation is associated with up-regulation of osteogenic markers in the *in vivo* 3D-scaffold explants

**Fig 4:**
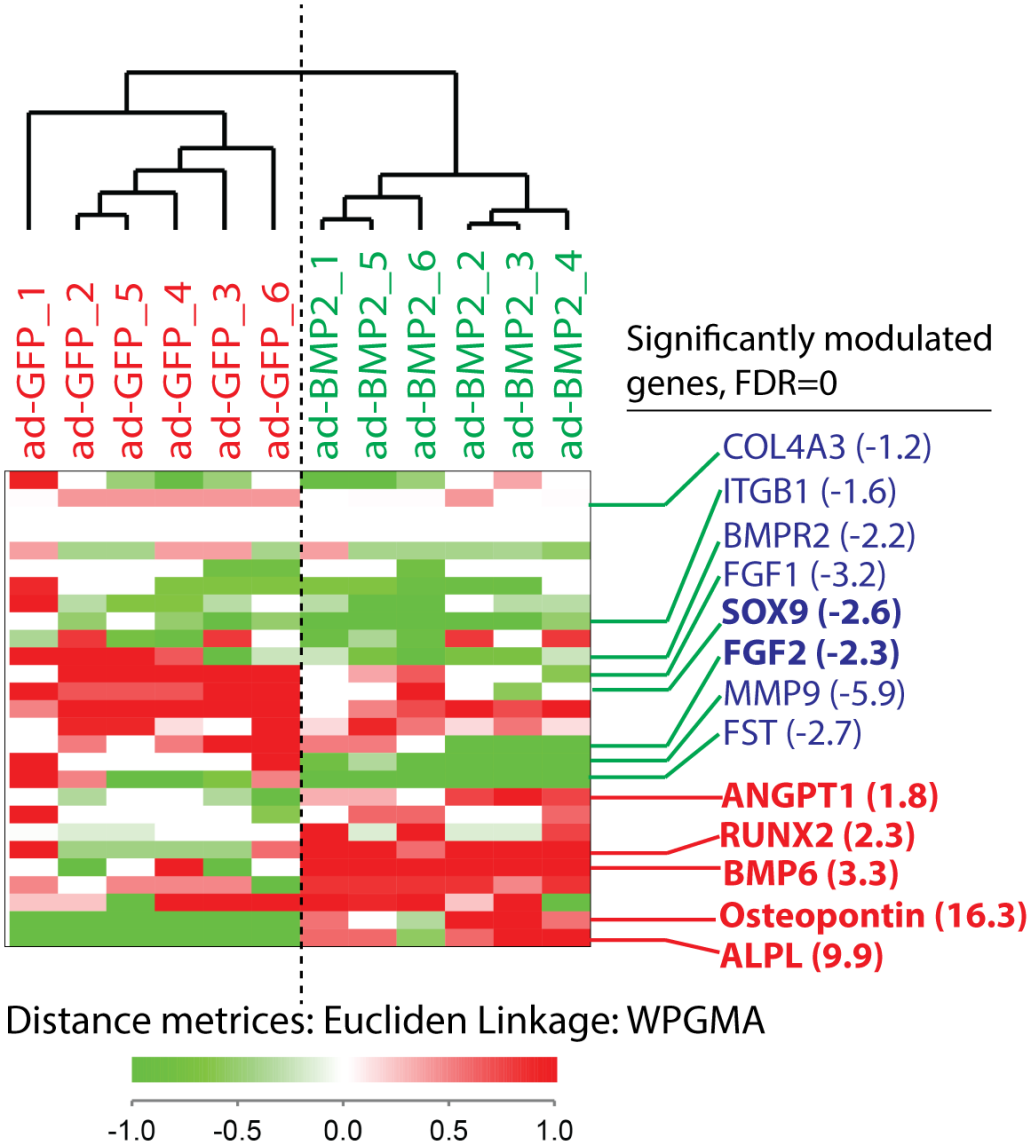
BMP2 over-expression mediated bone formation is associated with up-regulation of osteogenic markers in the in vivo scaffold explants. Custom PCR array was used to examine differentially expressed osteogenesis and angiogenesis related genes in scaffold explants at 2 weeks. Unsupervised hierarchal clustering demonstrated two separate clusters of the biological replicates (*n* = 6) from ad-BMP2 and ad-GFP BMSC groups. SAM analysis revealed significant up-regulation (FDR = 0) of a number of osteogenesis related genes such as *SPP1 (osteopontin)*, *ALPL*, *RUNX2*, *BMP6*, *ANGPT1* in ad-BMP2 explants as compared to the ad-GFP explants.

Custom PCR array was used to examine differentially expressed osteogenesis and angiogenesis related genes in 3D-scaffold explants at 2 weeks. Unsupervised clustering using the significantly modulated genes revealed two distinct clusters for ad-GFP and ad-BMP2 explants (Fig 4). SAM analysis showed significant (FDR = 0) up-regulation of osteogenesis related genes such as *SPP1* (16.3 fold change), *ALPL* (9.9 fold change), *BMP6* (3.3 fold change) *RUNX2* (2.3 fold change), in ad-BMP2 explants as compared to the ad-GFP explants (Fig 4). Additionally, *ANGPT1* (1.8 fold change), an angiogenic factor that modulates endothelial differentiation, was found to be significantly increased in ad-BMP2 explants (Fig 4). Of note, mRNA levels of *SOX9*, a key transcriptional factor required for the successive steps of chondrogenesis [27] was significantly down-regulated (2.6 folds) in ad-BMP2 explants (Fig 4). In parallel, mRNA levels of *FGF2*, an upstream positive regulator of SOX9 [28], was also under-expressed (2.3 folds) in ad-BMP2 explants (Fig 4).

### BMP2 over-expression mediated bone formation is not associated with enhanced chondrogenic activity

**Fig 5:**
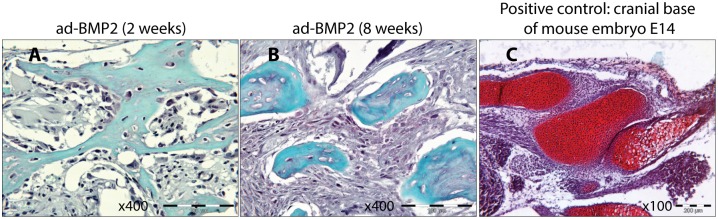
BMP2 over-expression mediated bone formation is not associated with enhanced chondrogenic activity. Formalin fixed paraffin embedded sections of ad-BMP2 explants at 2- and 8- weeks were stained with safranin O and counterstained with fast green. Bony structures in the scaffold explants (A and B) were negative for safranin O staining whereas the positive control (C, cranial base of mouse embryo E14) was intensely positive.

Significant down-regulation of *SOX9* and *FGF2* mRNA levels in ad-BMP2 explants led us to investigate the presence of cartilaginous extracellular matrix glycosaminoglycan by Safranin in the bony structures. No positive staining for Safranin O was detected in the bony structures in ad-BMP2 scaffold explants both at 2 and 8 weeks (Fig 5A and 5B). However, strong positivity was observed in the positive control (cranial base of mouse embryo E14) (Fig 5C).

## Discussion

Employing recently developed biodegradable poly(LLA-co-CL) scaffolds as a carrier, the current study examined the role of BMSC engineered to express BMP2 by adenovirus vector in bone formation. Our results demonstrated that adenoviral mediated BMP2 delivery significantly induced osteogenic differentiation of BMSC grown in poly(LLA-co-CL) scaffolds *in vitro* as well as bone formation ability *in vivo*.

Although the therapeutic importance of BMP2 in bone regeneration is well recognized, the precise and physiological delivery of BMP2 at the defect site is difficult to achieve [5]. It has been demonstrated that a short-term expression of the BMP2 is sufficient to irreversibly induce bone formation by BMSC [29], suggesting that a transient expression of BMP2 might be sufficient for cell-mediated BMP2 therapy for bone regeneration. These findings support the use of adenoviral mediated gene delivery system which has been shown to induce a transient (less than 2 weeks) gene expression *in vivo* [30]. In the current study, ad-BMP2 BMSC expressed significantly higher levels of *BMP2* mRNA and secreted protein (Fig 1), indicating that adenoviral mediated BMP2 transduction in BMSC was functional. PCR array showed significant up-regulation of several key osteogenic marker genes (*ALPL*, *RUNX2*, *BGLAP*, *BMP6*, *BMP7*) in ad-BMP2 at day 3 or day 14 *in vitro*. These results indicate that BMP2 was able to differentiate BMSC seeded in scaffolds (*in vitro*) towards an osteogenic pathway. Up-regulation of osteogenic marker genes was also observed in ad-BMP2 BMSC from donor 2 and 3 (S1 Fig), suggesting that osteogenic effects of BMP2 was not restricted to a limited type of BMSC strains. In addition to osteoinduction, angiogenesis is considered essential for bone regeneration [31]. Angiogenesis is a complex process requiring a coordinated interplay between growth factors, their receptor systems and down-stream signaling pathways [32]. In the present study, mRNA expression levels of *FLT1* (VEGFR1) and its ligands *VEGFA* and *PGF*, were significantly upregulated in ad-BMP2 BMSC respectively at day 3 and day 14 (Fig 2), suggesting that BMP2 expression was able to induce pre-angiogenic transcriptional program in BMSC. In line to these findings, previous studies have demonstrated up-regulation of VEGFA and PGF in pre-osteoblast like mouse cells [33] and human osteoblasts [34] with rhBMP2 treatment. Taken together, adenoviral mediated BMP2 expression in BMSC grown in poly(LLA-co-CL) scaffolds *in vitro* was able to induce osteogenic differentiation and to program BMSC towards more angiogenic genotype.

Examination of osteogenic ability of adenoviral mediated BMP2 expression was subsequently performed by subcutaneous implantation of poly(LLA-co-CL) scaffold seeded with BMSC in NOD/SCID mice. On gross visual examination, scaffold explants from ad-BMP2 group both at 2- and 8-weeks were reddish in color as compared to the explants from control groups (Fig 3B), indicating that these scaffold explants were richer in vascular supply. Supporting these observations, mRNA levels of *ANGPT1* (angiopoietin-1), which is crucial to the maturation of newly formed blood vessels, was significantly up-regulated in ad-BMP2 scaffold explants as compared to the ad-GFP control explants (Fig 4). This is in line with the *in vitro* results where BMP2 expression led to significant up-regulation of other pro-angiogenic molecules in ad-BMP2 BMSC (Fig 2 and S1 Fig). MicroCT analysis revealed no radiopaque bone like mass in any of the control groups, whereas a significant amount of radiopaque mass could be detected in the ad-BMP2 explants (Fig 3C–3G). Mirroring these results, histological examination of H&E stained sections showed no bony structures in the scaffold explants from any of the control groups, both at 2-and 8-weeks (Fig 3H–3M). In contrast, formation of bony structures could be identified in ad-BMP2 scaffold explant as early as 2-weeks, with more extensive bone formation by 8-weeks (Fig 3N and 3O). Numerous osteocytes like cells present in the bony structures in ad-BMP2 explants (Fig 3N and 3O, insets) suggested that the newly formed bone in the scaffolds was indeed a vital bone. Overall, these findings indicate that the adenoviral mediated expression of BMP2 in BMSC could induce formation of vital bony structures in poly(LLA-co-CL) scaffolds. Unsupervised hierarchical cluster analysis of the PCR array data showed a distinct mRNA profile of the genes related to osteogenesis and angiogenesis in ad-BMP2 explants as compared to ad-GFP explants (Fig 4), indicating a role for BMP2 in the regulation of these genes. SAM analysis further identified a number of osteogenic genes like *RUNX2*, *ALPL*, *SPP1* and *BMP6* to be significantly up-regulated in the ad-BMP2 explants. Collectively, these findings indicate that BMP2 enhances bone formation in poly(LLA-co-CL) scaffold seeded with ad-BMP2 BMSC by regulating the expression of key osteogenic genes such as *RUNX2*, *ALPL*, *SPP1* and *BMP6*.

Although some studies [35, 36] have suggested endochondral ossification as the mode for ectopic bone formation in the scaffold explants, the precise mechanism is currently unknown. In the current study, mRNA levels of *SOX9* and its upstream regulator, *FGF2*, was significantly under-expressed in ad-BMP2 explants at 2 weeks (Fig 4), suggesting that BMP2 mediated bone formation in poly(LLA-co-CL) scaffold explants was not related to enhanced chondrogenic activity. Supporting this suggestion, no positivity for safranin O staining was detected in bony structures in 2- and 8-weeks ad-BMP2 scaffold explants, whereas the positive control (cranial base of mouse embryo E14) was intensely positive (Fig 5). Hence, further studies are necessary to uncover the underlying mechanism of ectopic bone formation in scaffold explants.

In conclusion, results from the current study demonstrated that adenoviral mediated BMP2 delivery significantly induced mRNA expression levels of osteogenic and pro-angiogenic molecules *in vitro* in BMSC grown in recently developed, biodegradable poly(LLA-co-CL) scaffolds. Additionally, BMP2 over-expressing BMSC significantly enhanced bone formation in poly(LLA-co-CL) scaffolds in subcutaneous mouse model, with concomitant up-regulation of key osteogenic markers. Given the superior biodegradability, biocompatibility and mechanical properties of poly(LLA-co-CL) scaffolds and growing popularity of adenoviral vectors for gene therapy [37], their combination together with BMSC might, in future, be useful for bone regeneration therapy.

## Supporting Information

S1 Fig BMP2 induced in up-regulation of *ALPL*, *RUNX2* and *VEGFA* mRNA expression levels in ad-BMP2 BMSC from donor 2 and 3. mRNA levels of *BMP2* (A), *ALPL* (B and D), *RUNX2* (C and E), or *VEGFA* (F), were significantly over-expressed at day 3 or 14 in ad-BMP2 BMSCs from donors 2 grown in 3D scaffolds as compared to the control ad-GFP BMSC. mRNA levels of *BMP2* (G), *ALPL* (H and J), *RUNX2* (I and K), were significantly over-expressed at day 3 or 14 in ad-BMP2 BMSCs in ad-GFP BMSC from donors 3. Error bars represent SEM of 3 repeated experiments (*n* = 3) done in 3 technical replicates. ANOVA test with Bonferroni post hoc analysis was performed for statistical analysis. *** *P*<0.001; **P = 0.001–0.01.
(TIF)

S1 Text Supplementary Methods and Results. (DOCX)
