# Supplementary figures and images for: Adenoviral Mediated Expression of BMP2 by Bone Marrow Stromal Cells Cultured in 3D Copolymer Scaffolds Enhances Bone Formation
Source: PLoS One. 2016 Jan 25;11(1):e0147507. doi: 10.1371/journal.pone.0147507 (PMC4725849; doi:10.1371/journal.pone.0147507)

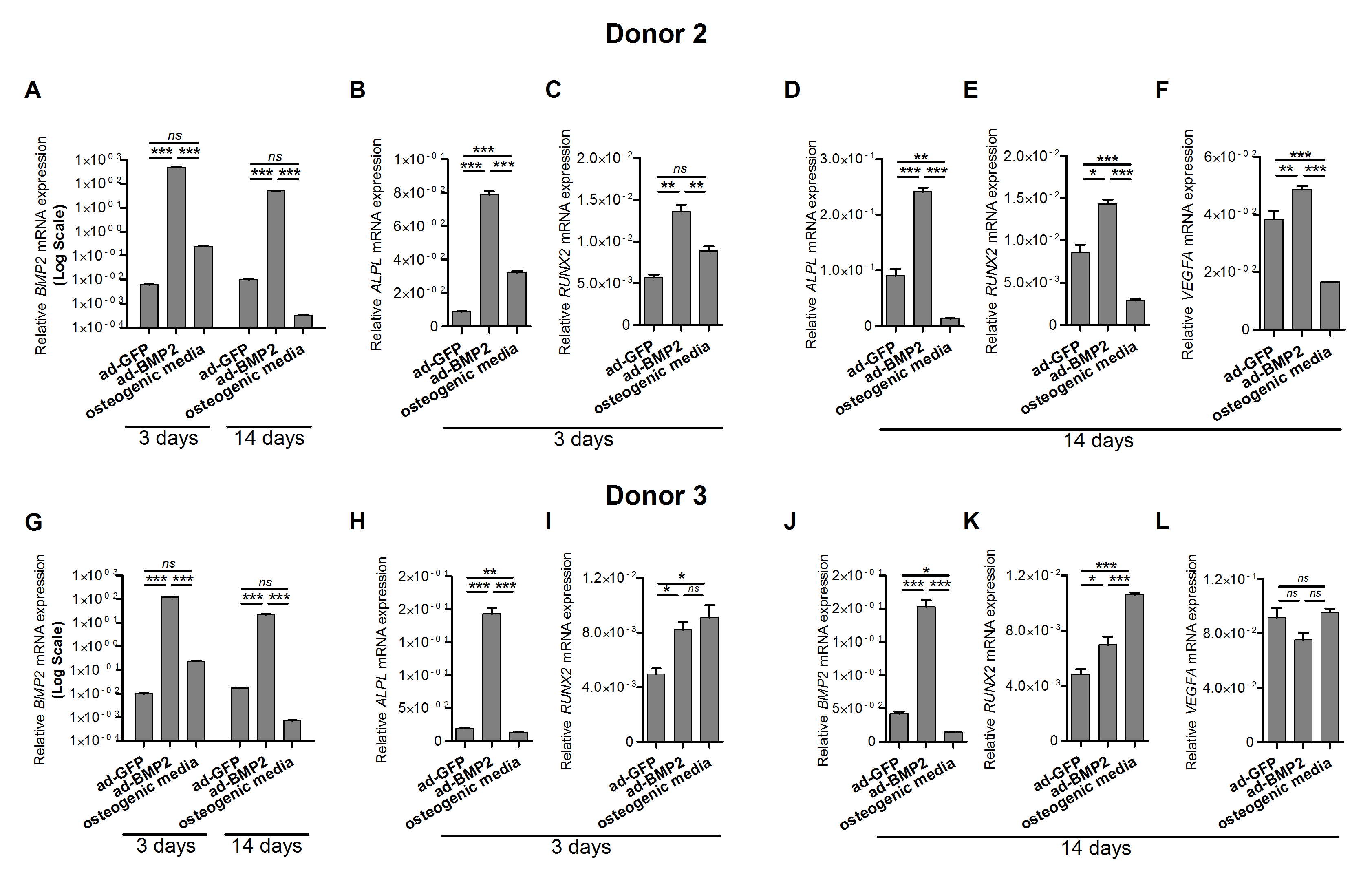

Supplement: S1 Fig — mRNA levels of BMP2 (A), ALPL (B and D), RUNX2 (C and E), or VEGFA (F), were significantly over-expressed at day 3 or 14 in ad-BMP2 BMSCs from donors 2 grown in 3D scaffolds as compared to the control ad-GFP BMSC. mRNA levels of BMP2 (G), ALPL (H and J), RUNX2 (I and K), were significantly over-expressed at day 3 or 14 in ad-BMP2 BMSCs in ad-GFP BMSC from donors 3. Error bars represent SEM of 3 repeated experiments (n = 3) done in 3 technical replicates. ANOVA test with Bonferroni post hoc analysis was performed for statistical analysis. *** P<0.001; **P = 0.001–0.01. (TIF) [file pone.0147507.s001.tif]
