# Supplementary material for: Adenoviral Mediated Expression of BMP2 by Bone Marrow Stromal Cells Cultured in 3D Copolymer Scaffolds Enhances Bone Formation
Source: PLoS One. 2016 Jan 25;11(1):e0147507. doi: 10.1371/journal.pone.0147507 (PMC4725849; doi:10.1371/journal.pone.0147507)
Supplement: S1 Text — (DOCX) [file pone.0147507.s002.docx]

**Methods**

For validation of the osteogenic and angiogenic gene expression changes induced by adenoviral mediated BMP2 expression in the donor 1 BMSC, human BMSC from two additional sources were used (henceforth referred to as donor 2 and 3 BMSC). Donor 2 BMSC were isolated, characterized and expanded from human bone marrow aspirates purchased from Lonza Walkersville Inc., MD, USA. The cells were characterized by flow cytometry, which showed that more than 90% of cells expressed stem cell surface marker of CD105, CD90, CD73 and less than 4% expressed CD34 and CD45 surface markers. Donor 3 BMSC were purchased from Lonza (Cat. number: PT-2501, Lonza Walkersville Inc., MD, USA). BMSC from donor 2 and 3 were grown in MSCGM^TM^ Mesenchymal stem cell growth medium (Cat. number: PT-3001, Lonza Walkersville Inc., MD, USA). As a positive control, BMSC from donors 2 and 3 were also grown in MSCGM^TM^ for 24 hours and replaced with osteogenic medium (MSCGM^TM^ plus 10^-8^M dexamethasone,50µg /ml ascorbic acid and 3.5mM β-Glycerophosphate).

**Results**

**Validation of BMP2 induced in up-regulation of *ALPL*, *RUNX2* and *VEGFA* mRNA expression levels in ad-BMP2 BMSC from donor 2 and 3.**

To confirm whether BMP2 induces expression of similar osteogenic and angiogenic genes in BMSC from other donors, we selected donors 2 and 3 BMSC, infected them with ad-BMP2 viruses and examined mRNA expression levels of *ALPL*, *RUNX2* and *VEGFA* by TaqMan qRT-PCR. BMSC grown in osteogenic differentiation medium served as positive controls. Similar to donor 1 BMSC, mRNA levels of *BMP2*, *ALPL*, *RUNX2* or *VEGFA* were significantly over-expressed at day 3 or 14 in ad-BMP2 BMSC from donors 2 and 3 grown in 3D scaffolds as compared to the control ad-GFP BMSC (S1 Fig). Of note, induction of these genes was significantly higher in ad-BMP2 BMSC as compared to that of osteo-BMSC (S1 Fig).
